# A journey mapping study of healthcare professionals’ involvement in fertility preservation for cancer patients

**DOI:** 10.3389/fonc.2026.1884218

**Published:** 2026-08-26

**Authors:** Di Xue, Aijun Zhu, Hongyuan Song, Meijuan Cheng, Zhenhua Lu, Huaijie Yang

**Affiliations:** 1 Affiliated Central People’s Hospital, China Three Gorges University, Yi Chang, China; 2 College of Medicine and Health Sciences, China Three Gorges University, Yi Chang, China; 3 Yichang Central People’s Hospital, Yi Chang, China

**Keywords:** cancer, fertility preservation, healthcare professionals, journey mapping, qualitative research

## Abstract

**Objective:**

Based on journey mapping, this study aimed to identify the main tasks, key pain points, and emotional experiences of healthcare professionals throughout their involvement in fertility preservation for cancer patients, and to provide evidence for designing targeted interventions and optimizing fertility preservation services.

**Methods:**

This qualitative study adopted purposive sampling to recruit 24 healthcare professionals from the departments of breast and thyroid surgery, oncology, and related departments at Yichang Central People’s Hospital between June and August 2025. Semi-structured interviews were conducted, and thematic analysis guided data coding and analysis. A journey map was then constructed.

**Results:**

The journey map of healthcare professionals’ engagement in cancer patients’ fertility preservation consisted of four stages: needs identification and topic communication, option assessment and referral, intervention and treatment, and long-term follow-up. Analysis of the interview data yielded 14 themes. The key pain points included insufficient knowledge, ambiguous role perception, the sensitivity of reproductive discussions, constrained medical resources, inadequate multidisciplinary collaboration, and gaps in follow-up care.

**Conclusion:**

Healthcare professionals acknowledge the importance of fertility preservation for cancer patients, yet they encounter multiple obstacles in clinical practice, such as deficiencies in professional knowledge and skills, weak interdisciplinary collaboration, and limitations in resources and policies. A multilevel intervention strategy should be developed through measures such as creating standardized assessment tools, building regional collaborative networks, strengthening knowledge and skill training with clear role definitions, improving cross-departmental information sharing, and prioritizing follow-up care. These efforts can enhance healthcare professionals’ engagement in fertility preservation and improve the quality of survival for cancer patients.

## 1 Introduction

Currently, cancer incidence is rising and the age of onset is trending younger (1). Data from the *2020 White Paper on the Quality of Life of Chinese Cancer Patients* show that the proportion of young patients (15–39 years old) continues to grow across many cancer types (2), and approximately 21% of female cancer patients are of reproductive age at diagnosis (3). Advances in cancer diagnosis and treatment have greatly improved survival for young patients, making post-treatment quality of life a key concern in comprehensive cancer care. Yet, the standard treatment modalities—surgery, radiotherapy, and chemotherapy—can severely impair or even irreversibly damage fertility while extending survival (4, 5). A total of 40% to 80% of patients face the risk of infertility (6, 7), and some have not yet married or had children, or still hold a desire for children at the time of diagnosis (8, 9). Furthermore, from 1990 to 2020, the average age at first childbirth for women in China moved from 23.43 years to 27.22 years (10). Fertility recovery research in other countries also indicates that, within the current medical context, the risks associated with first births before age 30 have declined considerably, but raising the probability of first births among those over 30 remains essential to maintain fertility rate stability (11). Therefore, both international and domestic sources highlight the necessity of addressing fertility preservation for reproductive-age cancer patients.

Fertility preservation refers to early prevention and treatment of factors that may impair male and female fertility, enabling individuals at risk of infertility to protect their reproductive potential, thereby safeguarding reproductive health and the ability to have biological offspring (12, 13). Research has found that 52.2% of cancer patients did not fully understand fertility risks and fertility preservation information before treatment (14, 15). During the decision-making process, patients face the dual pressure of survival and fertility. Limited decision time and lack of information often lead to decisional conflict or regret, which can affect their quality of life and psychological well-being (16). A gap, however, persists between clinical practice and guideline recommendations. The 2013 American Society of Clinical Oncology (ASCO) guideline on fertility preservation for cancer patients identified nurses as key members of the fertility preservation team (17), assigning them responsibility for facilitating referrals (18) and providing relevant knowledge and counseling (19). Meanwhile, the updated 2025 ASCO guideline explicitly states that, regardless of treatment gonadotoxicity or the patient’s sociodemographic characteristics, the care team should initiate fertility discussions throughout the entire treatment trajectory and make timely referrals to fertility clinics for preservation or to reduce psychological distress (20). In clinical settings, however, healthcare professionals do not always perform these roles effectively. Tight schedules may prevent physicians from conducting sustained, in-depth communication. Nurses, despite spending more time with patients and families and having a communication advantage, often face role ambiguity and knowledge gaps. Consequently, patients fail to receive timely key information and support. Studies show that up to 41.1% of healthcare professionals lack knowledge about fertility preservation (21). Existing research largely addresses the perspectives of patients or reproductive medicine technologies, but pays limited attention to healthcare professionals. Therefore, gaining a deeper understanding of the obstacles that healthcare professionals—especially nurses—encounter when delivering fertility preservation care is essential for meeting patient needs, implementing fertility decision aids, and improving healthcare quality.

A journey map is a visualization tool that visually and comprehensively presents the dynamic changes in emotions, interactions, barriers, needs, and other aspects (22, 23). Healthcare services have applied this tool to help healthcare professionals more fully acquire key diagnostic and management information. This study innovatively places healthcare professionals at the center, maps their journey of involvement in fertility preservation for cancer patients, identifies key pain points at each stage, and provides a basis for developing targeted intervention strategies.

## 2 Methods

### 2.1 Participants

We used purposive sampling to recruit healthcare professionals from the oncology department, thyroid and breast surgery department, gynecology department, and other relevant departments at Yichang Central People’s Hospital between June and August 2025. Participants met the following inclusion criteria: (1) holding a professional license and having at least two years of experience in cancer diagnosis, treatment, or nursing; (2) having contact with reproductive-age cancer patients; and (3) providing informed consent and volunteering to participate. We excluded interns, trainees, and those not currently on duty. Sampling continued until data saturation occurred, meaning no new themes emerged from the interviews, and we subsequently interviewed an additional 2–3 participants. We finally enrolled 24 participants, including 14 physicians (58.3%) and 10 nurses (41.7%). The departmental distribution was as follows: thyroid and breast surgery, 14 participants (9 physicians and 5 nurses); oncology department, 5 participants (4 physicians and 1 nurse); medical oncology, 1 participant (a physician); and gynecology, 3 participants (1 physician and 2 nurses). **Table 1** presents the participants’ basic characteristics. The hospital ethics committee approved this study (approval number: 2024-445-01).

**Table 1:** Basic information of the interviewed healthcare professionals (N = 24).

Department	Professional title	Sex	Age	Scope of practice	Highest education level	Working years
Thyroid and Breast Surgery	Chief Physician	Male	55	Licensed Physician	Bachelor	31
Thyroid and Breast Surgery	Associate Chief Physician	Male	57	Licensed Physician	Bachelor	33
Thyroid and Breast Surgery	Attending Physician	Male	35	Licensed Physician	Master	6
Thyroid and Breast Surgery	Associate Chief Physician	Male	42	Licensed Physician	Master	15
Thyroid and Breast Surgery	Associate Chief Physician	Female	51	Nursing	Associate degree	33
Thyroid and Breast Surgery	Supervisor Nurse	Female	47	Nursing	Associate degree	27
Thyroid and Breast Surgery	Supervisor Nurse	Female	36	Nursing	Bachelor	13
Thyroid and Breast Surgery	Supervisor Nurse	Female	29	Nursing	Bachelor	6
Thyroid and Breast Surgery	Supervisor Nurse	Female	29	Nursing	Bachelor	6
Thyroid and Breast Surgery	Chief Physician	Male	55	Licensed Physician	Bachelor	32
Medical Oncology	Chief Physician	Female	48	Licensed Physician	Doctor	22
Thyroid and Breast Surgery	Supervisor Nurse	Female	38	Nursing	Bachelor	16
Thyroid and Breast Surgery	Attending Physician	Male	30	Licensed Physician	Master	2
Oncology	Associate Chief Physician	Female	43	Licensed Physician	Master	16
Oncology	Chief Physician	Male	55	Licensed Physician	Master	33
Oncology	Attending Physician	Male	40	Licensed Physician	Master	14
Oncology	Supervisor Nurse	Female	33	Nursing	Bachelor	10
Thyroid and Breast Surgery	Attending Physician	Male	38	Licensed Physician	Doctor	12
Thyroid and Breast Surgery	Associate Chief Physician	Male	38	Licensed Physician	Doctor	12
Gynecology	Associate Chief Physician	Female	38	Nursing	Master	15
Gynecology	Associate Chief Physician	Female	51	Nursing	Bachelor	29
Gynecology	Chief Physician	Female	57	Licensed Physician	Bachelor	34
Thyroid and Breast Surgery	Associate Chief Physician	Male	55	Licensed Physician	Bachelor	32
Thyroid and Breast Surgery	Supervisor Nurse	Female	38	Nursing	Bachelor	16

### 2.2 Methods


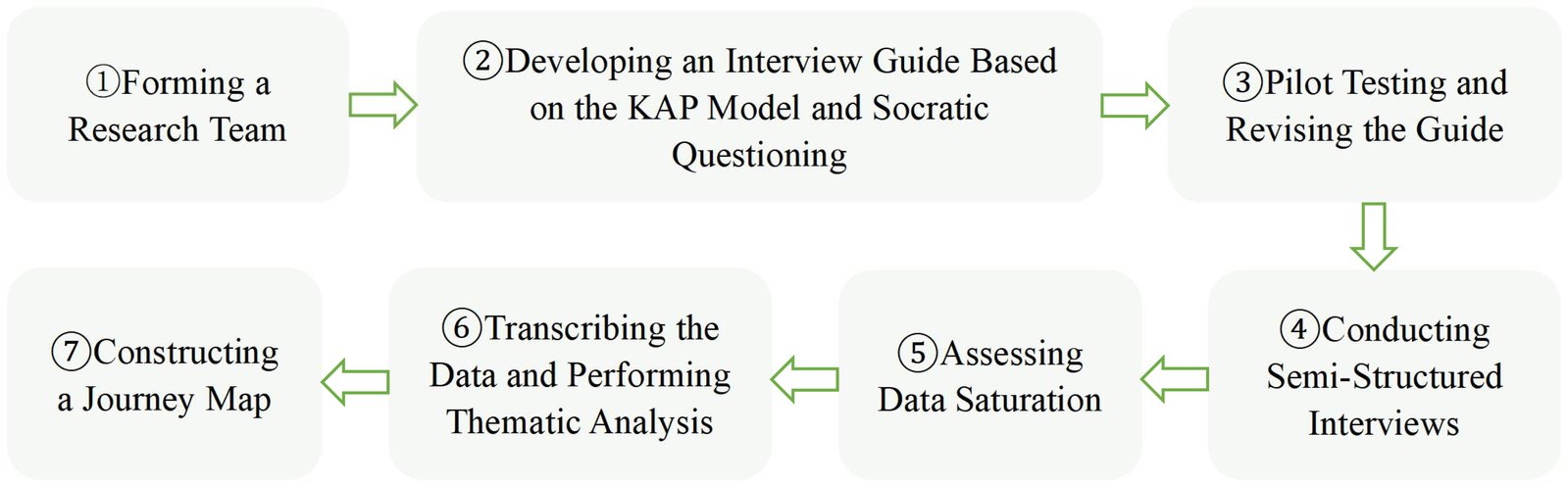


#### 2.2.1 Forming the research team

We formed a research team based on the study content. The team comprised two master’s students specializing in obstetric and gynecological nursing, one chief nurse in reproductive health, one head nurse of the thyroid and breast surgery department, and two graduate nursing students pursuing a master’s degree. The two master’s students conducted the interviews. The interviewers and the participants worked in the same hospital but in different departments; their relationship was one of equal collaboration, with no direct supervisory or administrative ties. The interviewers had no personal contact with the participants before the interviews. The chief nurse and head nurse provided overall guidance and supervision and did not directly participate in the interviews.

#### 2.2.2 Development of the interview guide

The interview guide was designed using the Knowledge, Attitude/Belief, Practice (KAP) model as the framework, with Socratic questioning serving as a scaffold. The KAP model is a commonly used framework for explaining how personal knowledge and beliefs influence behavioral change. This model divides behavioral change into three continuous processes: acquiring knowledge, generating beliefs, and forming behaviors. “Knowledge” refers to the recognition and understanding of relevant information, “belief” indicates correct convictions and positive attitudes, and “practice” denotes action. These three elements share a dialectical relationship: knowledge is the foundation for behavioral change, and beliefs and attitudes are the driving force. Only after individuals acquire sufficient relevant knowledge and form a personal understanding can they establish the belief needed for change, develop a positive psychological attitude, and ultimately guide their actions.

Socratic questioning guides critical thinking through a series of targeted questions, and its structured probing framework aligns closely with the purpose of the qualitative interviews in this study. The method mainly includes five types of questions: clarifying questions, which prompt individuals to articulate the core ideas or concepts of their views; probing questions, which guide individuals to analyze the logic behind their views in depth by exploring reasons; consequence questions, which predict and examine the possible outcomes or value of views; comparative questions, which encourage individuals to present views from opposing or different perspectives, thereby broadening the scope of the dialogue; and reflective questions, which foster self-reflection. When listing and ordering questions, the following sequence was used:

1. Definition and Concept: According to Socratic questioning, the first step is to clarify core concepts, unify the research context, avoid terminological ambiguity, and reveal differences in how healthcare professionals perceive “cancer reproductive care.” Within the KAP model, “knowledge” forms the basis for behavioral change; therefore, ensuring conceptual clarity at the very beginning of the interview is essential.
2. Assumptions and Preconceptions: This step aims to understand healthcare professionals’ personal subjective views and identify the preconceptions that influence their delivery of cancer reproductive care. In the KAP model, “belief” determines behavioral motivation, so personal preconceptions directly affect care behaviors.
3. Reasons and Evidence: This step explores the factors that influence behavior. In the KAP model, knowledge, beliefs, and the environment all shape behavior. After identifying personal subjective factors, the interview further examines environmental, objective, social, and cultural factors to distinguish individual knowledge gaps from systemic deficiencies.
4. Perspectives and Viewpoints: By recalling past experiences, this step prompts deeper reflection and reveals genuine feelings during actual clinical practice. Within the KAP model, personal experience also serves as a source of knowledge. Exploring the origins of obstacles and related cases can provide practical support for later strategy development.
5. Consequences and Implications: This step assesses the potential impact of the current situation, emphasizes the urgency of cancer reproductive care, and further explores healthcare professionals’ awareness of “fertility risks” for patients. Feedback on behavioral consequences can reinforce knowledge and beliefs, and raising hypothetical consequences helps stimulate the motivation for behavioral change.
6. Questions and Purpose: This step identifies the support healthcare professionals actually lack and the help they most need. Building on the preceding questions, it summarizes current practices and obstacles from various angles, ultimately grounding the discussion in clinical improvements.

#### 2.2.3 Pilot study

After drafting the initial interview guide based on the study content and objectives, we invited healthcare professionals from departments such as thyroid and breast surgery and obstetrics and gynecology to provide suggestions for refinement. We then conducted pilot interviews with two eligible healthcare professionals and adjusted the content and sequence of the guide based on their feedback, thereby finalizing the formal interview guide.

#### 2.2.4 Interview implementation

Researchers who had completed systematic training in qualitative research conducted this study. Immediately after each interview, the researchers recorded their subjective impressions and potential biases and regularly discussed them with research team members to minimize subjective bias and ensure procedural rigor. Interviewers contacted participants in advance to arrange a mutually convenient time and location. They selected quiet, private settings such as demonstration rooms to avoid interruptions and protect confidentiality. Before each interview, the researchers explained the study purpose, methods, and content in detail and assured participants that all interview content and personal information would remain strictly confidential, which helped build trust and obtain informed consent. After obtaining consent, the researchers audio-recorded the entire interview. Each interview lasted 30 to 60 minutes. During the interview, the researchers focused on the participants’ narratives, asked appropriate follow-up questions while avoiding leading or suggestive language, and observed and recorded nonverbal cues such as facial expressions, changes in tone, and body movements to ensure authentic data collection.

#### 2.2.5 Data saturation assessment

Following the principle of data saturation, the researcher conducted a preliminary analysis after each interview. Once no new themes emerged, another two to three interviews were conducted, and data collection then ended.

#### 2.2.6 Data organization and analysis

We analyzed the interview data using thematic analysis. Within 24 hours of each interview, we transcribed the recordings verbatim, added notes on nonverbal information, and had two researchers listen and cross-check the transcriptions for accuracy. Two other researchers then used NVivo 12 to assist with coding. They followed a sequence of data familiarization, extraction of key statements, open coding, extraction of recurring ideas, clustering of themes, refining core themes, and verification. The two researchers coded independently, and a third researcher resolved any disagreements. This study kept complete records, including interview recordings, transcripts, and documentation of the thematic coding process.

## 3 Results

**Figure 1:**
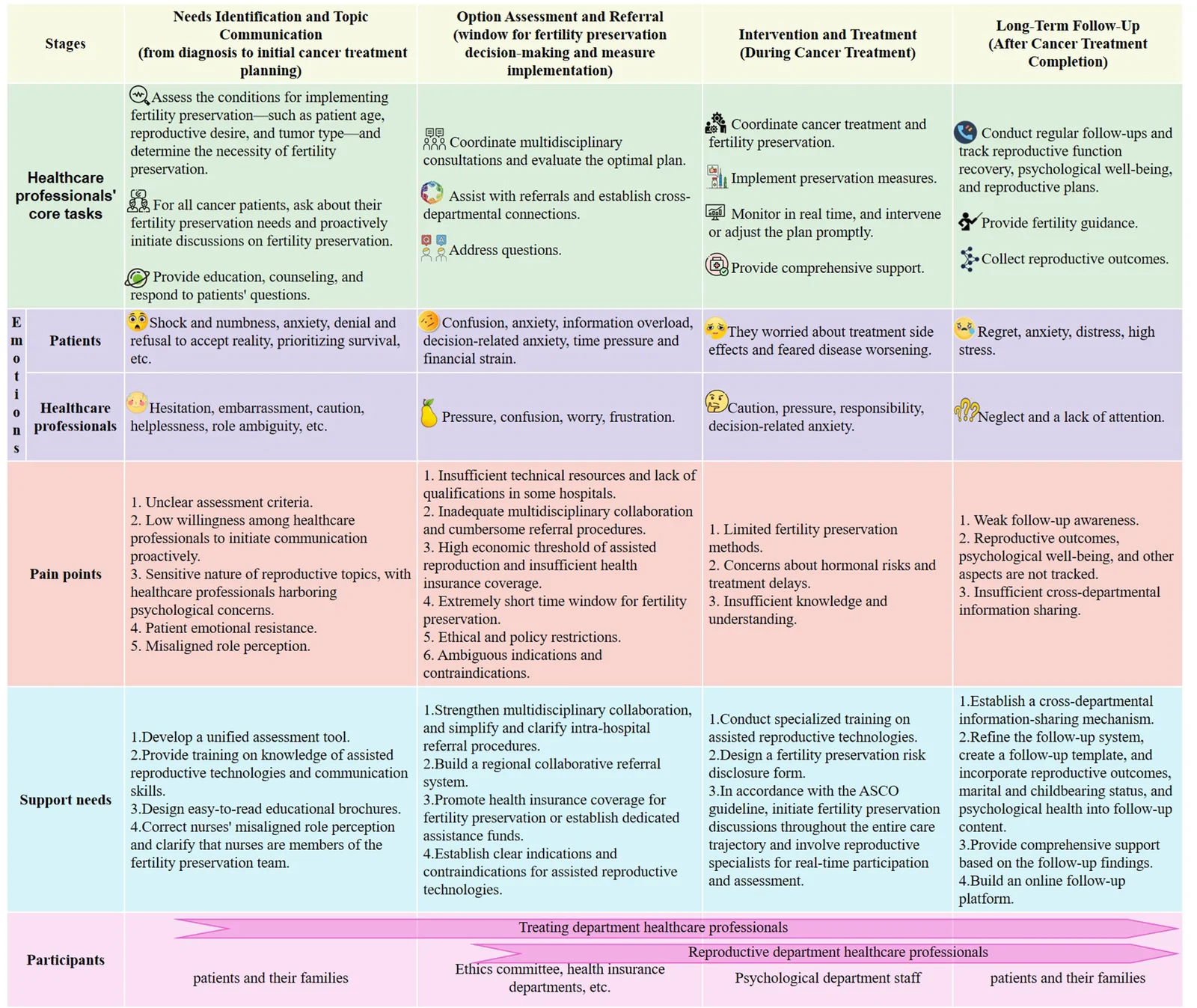
Journey map of healthcare professionals’ involvement in fertility preservation for cancer patients.

A journey map typically consists of a horizontal axis (timeline) and a vertical axis (task line). Drawing on the ASCO guideline’s recommendation to “initiate fertility discussions throughout the entire treatment trajectory” and the actual clinical workflow of cancer diagnosis and treatment, we categorized the themes into four consecutive stages. After repeated discussions, the research team finalized this categorization as the horizontal axis: Needs Identification and Topic Communication, Option Assessment and Referral, Intervention and Treatment, and Long-Term Follow-Up. The vertical axis, aligned with the study’s aim, included core tasks, emotions, pain points, and support needs. Based on this journey map framework, we conducted an in-depth analysis of the interview data. Two researchers primarily assigned the specific themes within each stage. Disagreements were resolved through group discussion and adjudication by a third researcher. After the preliminary map was formed, we invited two experts to review it and adjusted the theme labels and assignments according to their feedback. We did not conduct participant validation. Ultimately, we identified 14 themes across the four stages, capturing the main tasks, emotional fluctuations, and obstacles healthcare professionals encountered when making judgments at each stage of their involvement in fertility preservation. This process resulted in the journey map of healthcare professionals’ participation in fertility preservation, as shown in **Figure 1**.

### 3.1 Stage 1: needs identification and topic communication (from diagnosis to initial cancer treatment planning)

During this stage, healthcare professionals serve as the “trigger” for fertility preservation-related topics and content. In their communication with patients, these professionals assess the patient’s age, reproductive desire, tumor type, and other factors, determine the necessity of fertility preservation, proactively initiate related discussions, provide education and counseling, and respond to the patient’s questions.

#### 3.1.1 Pain points

1. The assessment standards and procedures for fertility preservation remain unclear, and no unified basis exists to guide the timing of discussions. Some healthcare professionals believe the approach should follow the patient’s reproductive needs. For example, one said, “We should ask anyone who has a reproductive need; if they have no such need, asking would be pointless.” Others consider age a key criterion for initiating fertility preservation discussions. One participant stated, “I think we need to divide patients by age. Those under 40 need it; those over 40 probably do not, though some may still want it.”
2. Healthcare professionals show low willingness to proactively identify patients who need fertility preservation. Their knowledge is insufficient, and they worry about being unable to answer patients’ questions, so they mainly rely on patients to raise the topic. Quotations include: “Most of them don’t bring it up on their own. Patients are dazed when they first hear the word ‘cancer’,” “Usually we don’t mention it unless the patient asks first; only then do we talk about it,” “We nurses don’t get involved in this area and don’t do related education,” and “Our knowledge is still lacking, and we’re afraid we won’t be able to answer if the patient asks, so we tend not to initiate the conversation.”
3. In China’s sociocultural context, reproductive topics are sensitive, especially after patients have just received a cancer diagnosis. Healthcare professionals harbor psychological concerns and generally do not ask proactively. As they explained, “Because this is a sensitive topic, we usually don’t bring it up on our own,” and “We’re afraid of invading the patient’s privacy and feeling awkward.”
4. In the early post-diagnosis period, patients experience emotional breakdown and resistance, making communication difficult and preventing rational consideration of fertility issues. Comments include: “Patients break down emotionally after diagnosis,” “Patients are most concerned about survival and have no mind to think about reproduction,” and “At first diagnosis, they are in denial and refuse to accept the cancer; at this point, they cannot make rational judgments about fertility preservation.”
5. Role perception is misaligned. Although guidelines clearly state that nurses should be part of the fertility preservation team, with responsibilities for facilitating referrals and providing relevant knowledge and counseling, some nurses believe that “nurses don’t interfere in this area and don’t do related education; this duty mainly falls on doctors,” and “It’s not really appropriate for nurses to ask about reproductive needs. The treatment plan is determined by doctors; this kind of professional question should be directed to doctors, not us.”

#### 3.1.2 Emotions

1. Healthcare professionals’ emotions: hesitation (worry that reproductive topics may trigger anxiety and psychological burden in patients), embarrassment (due to the sexual and reproductive nature of the topic), caution (uncertainty about patients’ emotions, needs, and receptivity), helplessness (patients’ resistance to communication after diagnosis), and role ambiguity (some nurses believe fertility preservation discussions should be led by physicians).
2. Patients’ emotions: shock and numbness (a psychological defense stage immediately after cancer diagnosis), anxiety (receiving a large amount of negative information in a short period), denial and refusal to accept reality, and prioritizing survival above other concerns.

### 3.2 Stage 2: option assessment and referral (after the fertility preservation decision is made and before measures are implemented)

At this stage, healthcare professionals shift to a “supporter” role. Their core tasks include establishing effective cross-departmental connections, evaluating the most suitable fertility preservation options for patients, coordinating multidisciplinary consultations, assisting patients with referrals, and addressing patient questions about options, costs, and other related concerns.

#### 3.2.1 Pain points

1. Some hospitals lack sufficient technical resources, and certain regions do not have the qualifications for assisted reproduction, requiring referral to higher-level hospitals. As interviewees stated, “Our hospital can’t do it, so we refer patients to a higher-level hospital,” and “Yichang doesn’t have the qualification; patients have to go to Tongji Hospital in Wuhan. That also involves ethics and qualification issues, and the cost is high.”
2. Multidisciplinary consultation is not routine practice, and the involvement of reproductive medicine departments remains low, which hinders interdisciplinary collaboration. Moreover, referrals—whether within the hospital, to other hospitals, or across regions—involve cumbersome procedures. As interviewees noted, “We do have multidisciplinary consultations, but they rarely cover fertility preservation,” and “We tell patients to go to the outpatient clinic and find the reproductive center.”
3. Assisted reproductive technologies carry a high economic threshold, and health insurance coverage remains insufficient. This further intensifies the financial strain on patients who already bear the costs of cancer treatment. As interviewees noted, “Things like egg freezing aren’t covered by insurance. Some places have included assisted reproduction in insurance, but we don’t have that here yet,” and “Insurance doesn’t work well—reimbursable items for assisted reproduction and family planning are extremely limited.”
4. The time window is extremely short. The urgency of cancer treatment affects decisions on fertility preservation and the implementation of certain assisted reproductive technologies. Additionally, ethical and policy restrictions further prolong the time needed to carry out these measures. As interviewees described: “Unmarried patients face restrictions on egg freezing,” “For unmarried individuals, our hospital in Yichang doesn’t have the qualification to freeze sperm or eggs; they have to go to Wuhan. For married patients, spousal consent is required—otherwise, freezing cannot proceed,” and “After diagnosis, patients may need surgery within one or two weeks and chemotherapy within a month—there is simply no time to fully understand and prepare for egg freezing.”
5. The lack of clear evidence leaves the indications and contraindications for assisted reproductive technologies in cancer patients ambiguous: “We don’t know the optimal timing for egg freezing before chemotherapy in breast cancer patients, and we worry it might compromise treatment,” “There’s no medical consensus, so in the end everyone just avoids extra trouble,” “The risks of egg freezing are uncertain, and it could delay treatment,” and “Doctors also fear tumor recurrence if the patient becomes pregnant, so they don’t dare give casual advice.”

#### 3.2.2 Emotions

1. Healthcare professionals’ emotions: Pressure (balancing cancer treatment with the timely provision of fertility preservation, while oncology staff already face heavy workloads and psychological stress), Confusion (unclear applicability criteria for fertility preservation options), Worry (concern that patients cannot proceed due to costs, ethical or policy restrictions, or that fertility preservation might compromise cancer treatment), Frustration (resource limitations or institutional gaps in certain regions or hospitals, insufficient personal knowledge, and unclear referral pathways, all hindering the delivery of comprehensive fertility preservation care and generating a sense of failure).
2. Patients’ emotions: Confusion (lack of decision-making aids, leaving them unable to judge whether fertility preservation will affect cancer treatment), Information overload (an overwhelming influx of online information, with no way to quickly verify its accuracy), Time pressure and financial strain.

### 3.3 Stage 3: intervention and treatment (during cancer treatment)

At this stage, healthcare professionals should act as implementers, coordinating the timing of cancer treatment and fertility preservation across disciplines, selecting the most suitable fertility preservation measures for the patient’s situation—such as administering pharmacological protection or assisting the reproductive department with assisted reproduction—while monitoring adverse effects during treatment and providing comprehensive support and counseling covering psychological care, cancer treatment, and fertility preservation.

#### 3.3.1 Pain points

1. Protective measures are rather limited, knowledge of emerging technologies is insufficient, and practice remains confined to conventional assisted reproductive methods, relying mainly on medication. As interviewees stated, “For example, we give goserelin to protect ovarian function, but we don’t know much about other options,” and “It’s mainly medication; very few people undergo egg or embryo freezing.”
2. Assisted reproductive technologies are infrequently used. Concerns about hormonal risks and treatment delays persist, and the potential conflicts between treatment and preservation remain unclear. Interviewees noted, “Egg freezing requires ovulation induction first, the cycle is long, and it involves hormones. Patients fear the hormones will worsen the cancer,” and “The risks of egg freezing are uncertain, and it would delay treatment. Doctors are also afraid of taking responsibility.”
3. Healthcare professionals lack adequate knowledge of fertility preservation and the details of assisted reproductive technologies. They expressed, “This is an interdisciplinary field, not my specialty,” “I’m afraid my knowledge is insufficient to explain it clearly,” and “It’s mainly that our own knowledge is lacking.”

#### 3.3.2 Emotions

1. Healthcare professionals’ emotions: Caution characterized their practice, as they mainly relied on conventional pharmacological protection and could not recommend a broader range of assisted reproductive technologies due to uncertain risks, limited hospital resources, and insufficient personal knowledge. Pressure arose from the need to balance the competing demands of treatment and preservation. A sense of responsibility made them concerned about intervention effectiveness and treatment safety. Decision-related anxiety emerged as they feared offering inappropriate advice and worried about potential risks and liability.
2. Patients’ emotions: They worried about treatment side effects and feared disease worsening.

### 3.4 Stage 4: long-term follow-up (after cancer treatment completion)

At this stage, healthcare professionals should focus primarily on monitoring the patient’s post-treatment recovery of reproductive function (e.g., ovarian or testicular function recovery), psychological well-being, and the fulfillment of reproductive plans. They should also conduct regular follow-ups, provide fertility guidance, and collect data on reproductive outcomes. Currently, however, healthcare professionals show weak follow-up awareness, and post-discharge follow-up remains inadequate. The patient’s psychological status, marital situation, and reproductive plans often go untracked. As interviewees noted, “Five to seven years after treatment, when the tumor recurrence rate drops, patients may want to marry and have children. That’s when they realize they didn’t preserve fertility, and they become anxious,” “It affects family, marital relationships, and psychological well-being,” and “Many young patients haven’t married or found a partner after treatment … but we have no data on whether the divorce rate is actually high.” One added, “Some patients’ marital relationships are affected.” Moreover, subsequent information exchange between the relevant departments and the reproductive department is lacking, and the long-term reproductive outcomes of patients who underwent fertility preservation often remain unavailable for various reasons. As an interviewee put it, “Some patients’ follow-up data just aren’t tracked.”

Therefore, healthcare professionals often experience neglect (viewing reproductive outcomes as a non-core tracking goal) and a lack of attention, while patients may suffer from regret, anxiety, distress, and high stress due to decisional regret, marital relationships, role identity crises, or recurrence risk.

## 4 Discussion

This study developed a four-stage journey map of healthcare professionals’ participation in fertility preservation: needs identification and topic communication, option assessment and referral, intervention and treatment, and long-term follow-up. The pain points across these stages clearly illustrate that although healthcare professionals recognize the value of fertility preservation, they face multiple obstacles in clinical practice—such as uncertainty about how to initiate communication, insufficient knowledge, and constraints related to medical resources, the environment, and the absence of clear standards. This finding aligns with a survey by Chen et al. among oncology nurses, which reported that nurses scored 77% on attitude toward fertility preservation, a lower 65.9% on knowledge, and only 37.2% on actual practice (18). Thus, while healthcare professionals demonstrate relatively positive awareness and attitudes, their knowledge, actions, and support lag behind—an issue that calls for urgent attention. A survey by Mousa et al. in Upper Egypt showed that as many as 92% of patients had low-to-moderate knowledge of fertility preservation, and 42.5% had never received any counseling on fertility and reproductive health from their medical team. The high unawareness rates for oocyte cryopreservation (71.5%) and embryo cryopreservation (74.8%) in that study (24) corroborate the pain points identified in our study, where nurses stated, “Our knowledge is still lacking … we’re afraid we won’t be able to answer if the patient asks.” Furthermore, analysis of the qualitative interview data revealed the influence of the Chinese sociocultural context. First, traditional childbearing beliefs, such as the imperative to “carry on the family line,” amplify the reproductive demands of some patients and their families and also intensify fertility-related anxiety. Under the dual pressures of survival and reproduction, patients and their families experience heightened emotional volatility, making it more difficult for healthcare professionals to initiate fertility discussions during the early post-diagnosis period. Second, sexual topics tend to be regarded as private matters in traditional culture, which triggers embarrassment and avoidance behaviors among healthcare professionals and further complicates communication. Therefore, intervention strategies must give full consideration to cultural factors.

### 4.1 Unifying assessment criteria and correcting role perception

During the needs identification and topic communication stage, the most prominent problem was the absence of a unified assessment tool and standardized guidelines. Additional barriers included healthcare professionals’ insufficient knowledge, limited understanding of the details and developments of assisted reproductive technologies, misaligned role perception, the sensitivity of the topic, and patient emotional distress. These factors collectively made it difficult for healthcare professionals to initiate fertility discussions proactively, which aligns with the pain points identified earlier. A scoping review by Tirsina et al. indicated that healthcare professionals’ knowledge of and attitudes toward fertility preservation stem primarily from career trajectory factors—such as their involvement in fertility preservation discussions with peers and patients, the number of patients they treat, and whether they have received advanced training—rather than from sociodemographic characteristics (25). This finding may explain, in part, the knowledge gaps and misaligned role perception observed among nurses in our study—namely, the lack of systematic training and a routine participation mechanism.

To improve this situation, the first step is to develop a unified assessment tool. The 2025 updated ASCO guideline recommends that clinicians ask about fertility preservation needs early, regardless of patients’ sociodemographic characteristics. Clinicians can then incorporate commonly used clinical criteria—such as age, marital and reproductive status, and fertility desire—to form a standardized assessment instrument.

Furthermore, healthcare professionals need to strengthen their knowledge of assisted reproduction. Particular attention should be given to defining the nurse’s role within the fertility preservation team and correcting the misperception that “nurses do not interfere.” Institutions can arrange regular lectures delivered by reproductive specialists and then implement tiered training. Basic knowledge of assisted reproductive technologies, the fertility preservation workflow, and relevant insurance and policies can be taught first. After professionals acquire sufficient foundational knowledge, communication skills training can follow, and scenario-based simulation used to consolidate knowledge and promote flexible application of communication techniques.

To help patients absorb information and alleviate healthcare professionals’ fear of embarrassment, hospitals can design easy-to-read educational brochures. Technical terms such as “ovarian tissue cryopreservation” and “oocyte cryopreservation” can be rewritten in patient-friendly language, and the brochures can list the fertility preservation process, indications, benefits, and risks. This approach strengthens patients’ and families’ understanding and facilitates subsequent communication. A survey by Martin et al. in Germany offered the patient perspective: although 72.9% of patients received fertility preservation counseling, only 56.1% received an offer of a preservation measure before treatment, and nearly half (49.5%) explicitly expressed a need for written information materials (26). This finding supports our intervention direction of designing educational brochures and further suggests that clinicians should offer counseling at multiple time points and in various forms, so that patients can obtain sufficient information within the urgent treatment decision-making window.

### 4.2 Streamlining referral processes and multidisciplinary collaboration

The barriers during the option assessment and referral stage stem largely from inadequate institutional support. Primary hospitals lack the necessary qualifications and equipment, the financial burden is heavy, and the narrow time window leaves only a short period for decision-making—all of which intensify the strain on patients. Moreover, critical questions such as “the optimal timing for egg freezing before chemotherapy” still lack a clear medical consensus, a gap that further fuels healthcare professionals’ avoidance behaviors. Furthermore, multidisciplinary consultations rarely address fertility preservation. The reproductive department seldom participates in treatment discussions for oncology patients, and patients must visit the reproductive center on their own. The absence of multidisciplinary collaboration and a clear referral process adds further obstacles to patients’ access to fertility preservation.

At this stage, institutions should build a regional collaborative referral system to clarify both intra-hospital and inter-hospital referral pathways. A referral procedure manual can compile contact information for reproductive centers and qualified facilities, ethical review processes, and fee schedules, allowing healthcare professionals to quickly access the necessary details. For intra-hospital referrals, a designated staff member should coordinate and track each case. For cross-regional referrals, a healthcare professional should first help the patient contact the receiving hospital and enable the sharing of medical records. Primary hospitals can also establish remote consultations through medical alliances.

Next, institutions should strengthen the multidisciplinary collaboration mechanism. The reproductive department needs to participate in treatment discussions for cancer patients who have fertility preservation needs. The relevant departments should take responsibility for formulating and implementing the cancer treatment plan and evaluating its compatibility with fertility preservation measures. The reproductive department should design the preservation plan, and nurses should handle communication, coordination, education, and follow-up. In addition, cross-department WeChat communication groups can facilitate timely discussions and prevent the situation where “multidisciplinary consultations never include fertility preservation.”

At the policy level, greater support is needed for investment in assisted reproductive technology equipment, qualification approval, and the scope of health insurance coverage.

### 4.3 Specialized technique training and clear delineation of risks

During the treatment stage, the pain points stemmed from limited knowledge and concerns about risks, which led to underutilization of assisted reproductive technologies and a preference for conservative pharmacological protection. Moreover, the lack of a clear and unified standard for the timing of treatment and preservation hindered proactive intervention. A quantitative study from South Korea found that nurses’ total fertility preservation knowledge score was only 15.46 ± 3.64 (out of 25), with the methods knowledge dimension scoring the lowest at 3.54 ± 1.19 (out of 6); only 19.5% of nurses knew that “oocyte cryopreservation is the best method for female fertility preservation.” (27). These objective data corroborate our interview findings that “it is mainly medication; very few people undergo egg or embryo freezing” and that healthcare professionals “had limited knowledge of other assisted reproductive technologies,” indicating that the detailed knowledge of assisted reproductive technology methods represents a widespread weakness and that specialized training should focus on this area.

Hospitals should organize regular, specialized training on assisted reproductive technologies so that clinicians gain a clear understanding of each technique’s target population and operational procedures. Before initiating fertility preservation, professionals need to explain the risks to patients in detail. Health professionals, patients, and family members should then engage in joint discussions and sign a fertility preservation risk disclosure form. Although no specific guideline currently prescribes the timing of fertility preservation measures, clinicians should follow the updated ASCO guideline, which recommends initiating fertility discussions throughout the entire care trajectory regardless of treatment gonadotoxicity and making timely referrals to a reproductive clinic, where a reproductive specialist can make the final determination.

### 4.4 Establishing comprehensive, whole-cycle information tracking and follow-up

During the post-treatment follow-up stage, healthcare professionals largely overlook fertility preservation outcomes. They generally prioritize cancer recurrence and survival rates as the core tracking targets while neglecting reproductive outcomes and not incorporating the recovery of reproductive function into follow-up content. After the treatment cycle fully ends, patients’ fertility needs receive little attention, which causes anxiety or affects their quality of life. Insufficient information sharing between the reproductive department and the relevant clinical departments creates a gap between patients’ cancer treatment information and their fertility preservation information.

To improve this situation, institutions should establish a cross-departmental information-sharing mechanism that makes patients’ fertility preservation measures, treatment plans, and follow-up data traceable and accessible, thus avoiding problems such as “post-referral information disconnection” and “inability to track after treatment completion.” Nurses can communicate regularly with the reproductive department or with patients and their families to understand patients’ fertility needs or progress. For example, if a patient wishes to conceive after treatment, a nurse can contact the reproductive department in advance, inform them of the patient’s previous treatment history, and facilitate tailored guidance. Furthermore, institutions should refine the follow-up system, create a fertility preservation follow-up template, specify key follow-up time points, and focus on tracking and recording reproductive function recovery, fertility plans, psychological health, and marital status. They can also rely on internet technology to build an online follow-up platform.

Furthermore, healthcare professionals should strengthen psychological support throughout the entire cancer treatment cycle and during subsequent follow-up. Many cancer patients currently experience reproductive concerns, defined as excessive worry about fertility and child-rearing among individuals who have not completed their family-building plans. This worry stems from the reproductive uncertainty caused by cancer and its treatment and from a lack of fertility-related information. The concerns include worry about fertility, personal health, partner relationships, pregnancy, acceptance of infertility, and children’s health and care. Therefore, clinicians should incorporate mental health assessments into follow-up and provide psychological counseling for patients who need it. A study by Hussien et al. found that psychological capital moderates the relationship between fertility preservation knowledge and tocophobia: among patients with low psychological capital, more knowledge may actually intensify the fear of childbirth, whereas among those with high psychological capital, knowledge may alleviate the fear (28). This finding suggests that simply providing knowledge and education cannot fully resolve patients’ psychological distress. The “psychological counseling” measure proposed in this study should therefore be combined with interventions that enhance patients’ psychological capital (such as resilience, optimism, hope, and self-efficacy) to manage reproductive concerns more effectively.

## 5 Conclusion

This study innovatively applied journey mapping to the process of healthcare professionals’ involvement in fertility preservation and clearly revealed specific pain points across four stages: unclear assessment criteria, ambiguous nurse role positioning, insufficient knowledge, and cultural sensitivity during the needs identification stage; resource and financial barriers and lack of multidisciplinary collaboration during the referral stage; limited methods and risk concerns during the treatment stage; and lack of fertility outcome tracking during the follow-up stage. Due to misaligned role perception and knowledge gaps, nurses failed to fully perform the role recommended by the guidelines.

Future efforts should develop a unified assessment tool and strengthen nurse training; establish a regional collaborative referral and multidisciplinary mechanism; conduct specialized training on assisted reproductive technologies and refine risk disclosure; and build a cross-departmental long-term follow-up system that integrates psychological support. These measures will improve the full-process fertility preservation service, effectively meet cancer patients’ reproductive needs, and enhance their post-treatment quality of life.

## 6 Limitations

This study has several limitations. As a single-center qualitative study, the sample came from only one prefecture-level tertiary hospital, which limits both regional and institutional coverage. The findings may not reflect the actual situations in different regions or at different levels of healthcare institutions. In addition, purposive sampling may have included healthcare professionals who were more concerned about fertility preservation or more willing to voice their views, introducing selection bias. Face-to-face interviews may also trigger social desirability bias, and some responses may lean toward socially acceptable directions. Furthermore, this study did not conduct member checking, so the analysis results were not verified by the participants. The study also did not systematically collect participants' prior fertility preservation training which may have introduced confounding effects from their experience and knowledge backgrounds on the results. In terms of professional roles, the participants were predominantly physicians and nurses, and the study lacked the perspectives of psychologists, reproductive medicine specialists, nursing assistants, and other roles. This may have omitted some potential barriers in multidisciplinary collaboration. Regarding cultural and systemic transferability, China’s cultural context—such as fertility beliefs and the sensitivity of sexual topics as private matters—hospital organizational models in which oncology and reproductive departments are relatively independent, and health insurance policies may have exerted specific influences on the findings. Caution is therefore warranted when directly applying the journey map and intervention recommendations from this study to other cultural contexts, healthcare institutions at different levels, or healthcare systems in other countries. Although the department scope of this study mainly covered breast, thyroid, gynecology, and medical oncology, the identified issues—such as communication barriers, lack of referral pathways, insufficient knowledge, and neglect of follow-up—may also exist in other specialties that affect fertility in young cancer patients, such as hematological oncology and urological oncology. Future research can extend the journey map framework and validate it in light of the characteristics of these specialties. Finally, this study relied solely on qualitative interviews and lacked quantitative data. Future studies should adopt multicenter, mixed-methods designs, include patients and their families, conduct dual-perspective research involving both healthcare professionals and patients, and follow up on long-term reproductive outcomes to more comprehensively evaluate intervention effects and enhance the generalizability of the conclusions.

## Data Availability

The raw data supporting the conclusions of this article will be made available by the authors, without undue reservation.

## References

[1] Sung H Ferlay J Siegel RL Laversanne M Soerjomataram I Jemal A . Global cancer statistics 2020: GLOBOCAN estimates of incidence and mortality worldwide for 36 cancers in 185 countries. CA Cancer J Clin. (2021) 71:209–49. doi: 10.3322/caac.21660 33538338

[2] China Anti-Cancer Association . Why is cancer occurring at increasingly younger ages? (2023). Available online at: http://www.caca.org.cn/system/2023/10/23/030052345.shtml (Accessed January 30, 2026).

[3] Pang Y Mu C Feng R Fan Z Ding Q Yue Q . Factors influencing fertility preservation decision-making among female cancer patients: a mixed methods systematic review. J Nurs Sci. (2025) 40:34–9. doi: 10.3870/j.issn.1001-4152.2025.21.034

[4] Zaami S Montanari Vergallo G Moscatelli M Napoletano S Sernia S La Torre G . Oncofertility: the importance of counseling for fertility preservation in cancer patients. Eur Rev Med Pharmacol Sci. (2021) 25:6874–80. doi: 10.26355/eurrev_202111_27235 34859849

[5] Lambertini M Del Mastro L Pescio MC Andersen CY Azim HA Jr Peccatori FA . Cancer and fertility preservation: international recommendations from an expert meeting. BMC Med. (2016) 14:1. doi: 10.1186/s12916-015-0545-7 26728489 PMC4700580

[6] Moravek MB Confino R Lawson AK Smith KN Kazer RR Klock SC . Predictors and outcomes in breast cancer patients who did or did not pursue fertility preservation. Breast Cancer Res Treat. (2021) 186:429–37. doi: 10.1007/s10549-020-06031-4 33392838

[7] Adams E Hill E Watson E . Fertility preservation in cancer survivors: a national survey of oncologists' current knowledge, practice and attitudes. Br J Cancer. (2013) 108:1602–15. doi: 10.1038/bjc.2013.139 23579214 PMC3668471

[8] Sun M Lu Q Zhang P Ke S Liu C . Barrier factors and needs for medical staff assisting breast cancer patients in fertility preservation decision-making: a qualitative study. Chin Nurs Manage. (2025) 25:375–80.

[9] Wu K Chen Y Jin Y Zhang P An P . Investigation on fertility of young women with early breast cancer. Chin J Pract Surg. (2021) 41:1262–8. doi: 10.19538/j.cjps.issn1005-2208.2021.11.15

[10] Yang T Shi R . The construction of a fertility service system under the background of delayed childbearing. Population Econ. (2025), 31–43. doi: 10.3969/j.issn.1000-4149.2025.04.003

[11] Winkler-Dworak M Pohl M Beaujouan E . Scenarios of delayed first births and associated cohort fertility levels. Demography. (2024) 61:687–710. doi: 10.1215/00703370-11315685 38785350

[12] National Health Commission Of China . Reply to recommendation no. 7031 of the third session of the thirteenth national people's congress (2021). Available online at: https://www.nhc.gov.cn/wjw/jiany/202102/af70bc1c49024a3cbfb0bc7e9c42218f.shtml (Accessed January 30, 2026).

[13] Huang G . Overview of fertility preservation. J Pract Obstetrics Gynecology. (2016) 32:241–2.

[14] Sun M Lu Q He X Wang W Bian Y Liu C . Role and responsibilities of oncofertility nurse navigators in fertility protection of cancer patients: a scoping review. Chin Nurs Res. (2025) 39:4065–70. doi: 10.12102/j.issn.1009-6493.2025.23.022

[15] Li J Li Y Li M Zhao X Guan Y Liu Y . Investigation and research on the cognition of the fertility preservation of the oncology and reproductive related physicians and the patients with cancer. Chin J Family Plann. (2024) 32:1009–16. doi: 10.3969/j.issn.1004-8189.2024.05.006

[16] Jones G Hughes J Mahmoodi N Smith E Skull J Ledger W . What factors hinder the decision-making process for women with cancer and contemplating fertility preservation treatment? Hum Reprod Update. (2017) 23:433–57. doi: 10.1093/humupd/dmx009 28510760

[17] Loren AW Mangu PB Beck LN Brennan L Magdalinski AJ Partridge AH . Fertility preservation for patients with cancer: American Society of Clinical Oncology clinical practice guideline update. J Clin Oncol. (2013) 31:2500–10. doi: 10.1200/JCO.2013.49.2678 23715580 PMC5321083

[18] Chen X Kan Z Gu C Fu Y Yan X Yan L . Current situation and influencing factors of nurses' knowledge,attitude and practice on fertility preservation of female cancer patients in a cancer hospital. Chin J Nurs. (2024) 59:1490–6.

[19] Lubejko BG Bellfield S Kahn E Lee C Peterson N Rose T . Oncology nurse navigation: results of the 2016 role delineation study. Clin J Oncol Nurs. (2017) 21:43–50. doi: 10.1188/17.CJON.43-50 28107327

[20] Su HI Lacchetti C Letourneau J Partridge AH Qamar R Quinn GP . Fertility preservation in people with cancer: ASCO guideline update. J Clin Oncol. (2025) 43:1488–515. doi: 10.1200/JCO-24-02782 40106739

[21] Ye Y Chen A Liu Z . Investigation of present situation and influence factors of fertility preservation cognition in hospital staffs. J Reprod Med. (2020) 29:1603–11.

[22] Zhang H Wu L Hu X Tang Z Liu Y . Development of a patient journey map for sedated children receiving medical procedures in outpatient clinics. J Nurs Sci. (2025) 40:41–6. doi: 10.3870/j.issn.1001-4152.2025.19.041

[23] Zong X Wang J Zheng Z Wu F Huang Q Zhang W . An overview of patient journey mapping methodology and reporting recommendations: a qualitative research example. J Nurses Training. (2025) 40:2241–7. doi: 10.16821/j.cnki.hsjx.2025.21.001

[24] Mousa O Jaramillo J Alghanem M . Fertility preservation awareness among oncology patients: influencing factors. Egyptian J Health Care. (2025) 16:1147–58. doi: 10.21608/ejhc.2025.440628

[25] Tirsina A de Freitas C Silva S . Awareness and attitudes toward fertility preservation among healthcare providers: a scoping review of quantitative evidence. Hum Reprod Update. (2025) 31:497–511. doi: 10.1093/humupd/dmaf014 40580925

[26] Martin M Sehouli J Altmann J Stark E Karaman M Busse A . Fertility preservation counseling in reproductive-age women with cancer. Int J Gynecological Cancer. (2026) 36:104660. doi: 10.1016/j.ijgc.2026.104660 41974152

[27] Kim M Nho J Lee A . Oncology nurses' knowledge regarding fertility preservation for patients with cancer. Korean J Adult Nurs. (2019) 31:315. doi: 10.7475/kjan.2019.31.3.315

[28] Hussien SM Zoromba MA El-Gazar HE Mousa O Amin SM Atta MHR . Fertility-preservation knowledge, perceived barriers, and tokophobia among female oncology patients: the moderating role of psychological capital. SAGE Open Nurs. (2025) 11. doi: 10.1177/23779608251376079 41000248 PMC12457756

